# Facts about Apollinaris

**Published:** 1904-09

**Authors:** 


### Facts About Apollinaris.

In France, England, Germany and the United States, mineral spring waters play an important part in the daily economy; in each of these countries the sale of apollinaris is larger than it ever was before, and according to the London *Lancet* thirty millions of bottles of this water are consumed each year. The *Lancet,* which is recognised everywhere as the highest authority on matters pertaining to health, recently sent one of its special commissioners to the apollinaris spring, which is at Neuenahr, Germany, in order to place before the medical profession an authoritative description of the bottling of this water.

The report of this commissioner, published in the *Lancet* of January 30, 1904, shows that he was given access to every nook and corner of the vast establishment in which the apollinaris spring is situated, and that he saw from beginning to end the process by which the water is bottled there, ready for delivery to all parts of the world. This report is full of interesting scientific details describing the methods of bottling, and shows that the purity and healthfulness of the apollinaris water as it comes from the spring is preserved; it says that the chemical analyses which he made are in accord with those given at various times by the late Professor Virchow, Professor Liebreich, the late Sir Edward Frankland and other authorities, and that the water wherever it is bought is identical with the water taken direct from the spring, as apollinaris is bottled only at the spring and only with its own natural gas. The supply of both water and gas from the spring is considerably in excess of the present enormous demands.

